# Antifungal Activity of Plant Extracts against *Candida* Species from Oral Lesions

**DOI:** 10.4103/0250-474X.49128

**Published:** 2008

**Authors:** K. Prabhakar, L. Sathish Kumar, S. Rajendran, M. Chandrasekaran, K. Bhaskar, A. K. Sajit Khan

**Affiliations:** Department of Microbiology, Rajah Muthiah Medical College and Hospital, Annamalai University, Annamalai Nagar-608 002, India; 1 Department of Microbiology, Rajah Muthiah Medical College and Hospital, Annamalai University, Annamalai Nagar-608 002, India; 2 Department of Botany, Annamalai University, Annamalai Nagar-608 002, India

**Keywords:** Antifungal activity, candidiasis, seaweed

## Abstract

Seventy five patients with oral lesions attending the different departments of Rajah Muthiah Medical College and Hospital, Annamalai University were screened for *Candida*. Forty six (61.3%) *Candida* strains were isolated from the oral lesions. Of the 46 *Candida* strains, *Candida albicans* accounted for 35 (76.08%), *Candida glabrata* for 5 (10.86%), *Candida tropicalis* and *Candida krusei* for 2 (4.34%) each and *Candida parapsilosis* and *Candida guilliermondii* for one (2.17%) each. Antifungal activity of ethanol extracts of five plant species that included *Syzygium jambolanum, Cassia siamea, Odina wodier, Momordica charantia* and *Melia azedarach* and two algal species, *Sargassum wightii* and *Caulerpa scalpelliformis* were tested against 25 isolated strains by disc diffusion method. Antifungal activity was observed at 100 mg/ml for *Syzygium jambolanum, Cassia siamea* and *Caulerpa scalpelliformis* and at 10 mg/ml for *Sargassum wightii*.

Candidiasis is an acute or chronic, superficial or deep infection with a very wide clinical spectrum. Candidiasis occurs mostly in patients who are predisposed to an overgrowth of their own yeast flora. Oropharyngeal candidiasis occurs in patients with diabetes mellitus, those receiving antibacterial antibiotics and those infected with HIV 1 or HIV 2^1^.

Either nystatin suspension or the clotrimazole douches is the drug of choice in candidiasis for non-immunosuppressed adults. Patients with advanced HIV infection or other immunosuppressed disorders may not respond to clotrimazole and may require systemic therapy with ketoconazole, given 200 mg once daily or fluconazole given 100 mg once daily^1^.

Traditional herbal cures for superficial candidiasis is topical application using calendula and commiphora^2^. The antifungal properties of various phytoalexins that are naturally occurring antimycotic secondary metabolites are well researched but mainly for crop plants^3^,^4^ and relatively little work has been done on their medical applications. In a study carried out to determine the antifungal activity of the marine algae of the French Mediterranean Coast it was found that of the thirty one species investigated, eight species exhibited antifungal activity^5^. Very little research has been done on the antimycotic nature of whole plant remedies and the action of some herbs is rather speculative.

Seventy five patients attending the different departments of Rajah Muthiah Medical College and Hospital were screened for *Candida* species. Plain cotton tipped swabs was collected from oral lesions of the patients with diabetes mellitus and HIV/AIDS patients. The specimens were examined by Gram's stain and were inoculated in Sabouraud's dextrose agar with chloramphenicol. *Candida* species was isolated and identified based on sugar fermentation tests^1^, germ tube test^6^ and chlamydospore formation^6^.

The bark of *Syzygium jambolanum* DC (Myrtaceae), *Cassia siamea* Lamk. (Leguminaceae), *Odina wodier* Roxb. (Anacardiaceae) and the leaves of *Momordica charantia* Linn (Cucurbitaceae) and *Melia azedarach* Linn (Meliaceae) were collected during October 2002 from Annamalai University campus and *Sargassum wightii* (Sargassaceae) and *Caulerpa scalpelliformis* (Caulerpaceae) were collected during October 2002 from the Rameshwaram Coast (9° 45' N and 79° 0' E). The plants were identified in the Department of Botany, Annamalai University, where the herbarium was located.

The bark and leaves of the plants, and the marine macro algae were washed with tap water and surface sterilized with 10 per cent sodium hypochlorite solution. The samples were rinsed with sterile distilled water and air dried in shade at room temperature. The samples were ground into a fine powder and extracted in 70% aqueous ethanol as described by Vanden Berghe *et al*^7^. The solvent from the extract was removed under pressure at 40°. The solid was used in antifungal assay after dissolving in 5% aqueous DMSO.

Twenty five *Candida* strains that include 15 strains of *C. albicans* and 10 other strains of *Candida* species isolated from the oral lesions were subcultured and the turbidity was adjusted to 0.5 McFarland tube. The broth culture was plated on a Sabouraud's dextrose agar plate. Plant extracts were diluted at a concentration of 500, 250, 100 and 10 mg/ml in 5% aqueous DMSO. Whatman No. 1 filter paper discs were prepared, sterilized and impregnated with 20 μl of the extract. The discs were placed on the plates inoculated with *Candida* strains. The plates were incubated and the results were read on the following day. A zone of inhibition was considered as having antifungal activity. Five per cent aqueous DMSO was used as negative control. Fluconazole was used as a standard. Pharmaceutical preparation of fluconazole was obtained and the discs were impregnated with 25 μg of fluconazole and the susceptibility of *Candida* species was determined based on a zone size of equal or more than 18 mm^8^.

From the 75 patients screened, 46 strains of *Candida* were isolated. This included 35 (76.08%) strains of *C. albicans*, 5 (10.86%) strains of *C. glabrata*, 2 (4.34%) strains each of *C. tropicalis* and *C. krusei* and one (2.17%) strain each of *C. parapsilosis* and *C. guilliermondii*. Of the 67 patients with diabetes mellitus screened, 39 (58.2%) were positive for *Candida* and of the 8 patients with HIV screened, 7 (87.5%) were positive for *Candida*.

Candidiasis is an opportunistic infectious disease caused by the genus *Candida*, which includes eight species. The most common species of the *Candida* is *C. albicans*. The preponderance of isolation of *Candida* from the oral lesions in our study was *C. albicans* (76.08%) and our study conforms with the isolation of the *C. albicans* by the different investigators. People with poor oral hygiene may have a higher rate of oral yeast carriage. Also oral yeast carriage rates are generally higher in the patients receiving medical attention^1^. The patient population of the study is exclusively those who have come to our hospital for some ailment or the other.

HIV is an important predisposing factor for oral candidiasis. It was observed that candidiasis was as high as 87.5% among HIV patients. Though the number studied was small, the isolation rate of *Candida* was still considerable. In advanced HIV infection, the appearance of oropharyngeal candidiasis is a predictor of clinical progression to AIDS^9^. Moreover, nearly all patients who progress to clinical AIDS have oral candidiasis at some time^10^.

As per our study candidiasis has been associated with 58.2% of patients with diabetes mellitus. Diabetes mellitus is an important predisposing factor for candidiasis^11^. Other predisposing factors for candidiasis are AIDS, preceding surgery, iatrogenic immunosuppression, intravenous catheters, prolonged administration of antimicrobial agents, cytoreductive chemotherapy, neutropenia, hematologic malignant diseases, burns and intravenous drug abuse^1^.

We have observed that *S. jambolanum* and *C. siamea* have exhibited anticandidial activity at 100 mg/ml and *O. wodier* at 500 mg/ml (Table 1). Extracts of *M. charantia* and *M. azedarach* have not exhibited any activity and so their activity has not been shown in the table. The algal extracts of *S. wightii* and *C. scalpelliformis* have also exhibited anticandidial activity at 10 and 100 mg/ml, respectively (Table 1). The zone of inhibition produced by the 70% aqueous ethanol extracts of bark of *S. jambolanum*, *C. siamea* and *O. wodier* and the algal extracts of *S. wightii* and *C. scalpelliformis* by agar diffusion test against all the 25 isolates of *Candida* species ranged from 7 to 22 mm. Fluconazole positive control produced a zone of inhibition of 18 mm or more on all the 25 isolates of *Candida* species tested. Five per cent aqueous DMSO negative control did not have any inhibitory effect on any of the 25 isolates of *Candida* species tested (Table 1).

**TABLE 1:** ANTIFUNGAL ACTIVITY OF SOME PLANT EXTRACTS AGAINST 25 ISOLATES OF *CANDIDA SPECIES*

*Candida species*	*S. jambolanum*				*C. siamea*				*O. wodier*				*S. wightii*				*C. scalpelliformis*			
					
	500	250	100	10	500	250	100	10	500	250	100	10	500	250	100	10	500	250	100	10
	(mg/ml)				(mg/ml)				(mg/ml)				(mg/ml)				(mg/ml)			
*C. albicans* (15)^a^	4+	3+	2+	-	4+	3+	2+	-	2+	-	-	-	4+	3+	2+	-	4+	3+	2+	-
*C. glabrata* (4)	4+	3+	2+	-	4+	3+	2+	-	2+	-	-	-	4+	3+	2+	-	4+	3+	2+	-
*C. tropicalis* (2)	4+	3+	2+	-	4+	3+	2+	-	2+	-	-	-	4+	3+	2+	-	4+	3+	2+	-
*C. krusei* (2)	4+	3+	2+	-	4+	3+	2+	-	2+	-	-	-	4+	3+	2+	-	4+	3+	2+	-
*C. parapsilosis* (1)	4+	3+	2+	-	4+	3+	2+	-	2+	-	-	-	4+	3+	2+	-	4+	3+	2+	-
*C. guilliermondii* (1)	4+	3+	2+	-	4+	3+	2+	-	2+	-	-	-	4+	3+	2+	-	4+	3+	2+	-

a indicates number of isolates; - indicates no activity; 1+ indicates zone of inhibition in average of 7 to 10 mm; 2+ indicates zone of inhibition in average of 11 to 14 mm; 3+ indicates zone of inhibition in average of 15 to 18 mm; 4+ indicates zone of inhibition in average of 19 to 22 mm.

The zone of inhibition recorded at 500 mg/ml concentration was higher than that of 250, 100, 10 mg/ml concentration for all the extracts. As the amount of the extract increased, the inhibitory effect has also increased. Similar findings were observed by Perumalsamy *et al.*^12^

*Candida* is a difficult organism to establish standards of therapeutic activity. The problem is compounded for plant extracts in which variation may be expected between samples depending on genotype, area of cultivation, time of harvesting, processing methods, dilution etc. The extracts in our study are crude and that may be the reason for the antifungal activity of these extracts up to 100 mg/ml for the *S. jambolanum, C. siamea* and *C. scalpelliformis.* Probably, a more refined preparation would have antifungal activity at a lower concentration. Further experimental and clinical trials should be carried out and if these antifungals are effective they could be used as therapeutic agents.
